# Evaluation of an automated knowledge based treatment planning system for head and neck

**DOI:** 10.1186/s13014-015-0533-2

**Published:** 2015-11-10

**Authors:** Jerome Krayenbuehl, Ian Norton, Gabriela Studer, Matthias Guckenberger

**Affiliations:** Department of Radiation Oncology, University Hospital Zurich, Klinik für Radio-Onkologie, Rämistrasse 100, CH-8091 Zürich, Switzerland; Philips Radiation Oncology Systems, Fitchburg, WI USA

**Keywords:** Volumetric modulated arc therapy, Head and neck, Automated planning optimization

## Abstract

**Background:**

This study evaluated an automated inverse treatment planning algorithm, Pinnacle Auto-Planning (AP), and compared automatically generated plans with historical plans in a large cohort of head and neck cancer patients.

**Methods:**

Fifty consecutive patients treated with volumetric modulated arc therapy (Eclipse, Varian Medical System, Palo Alto, CA) for head and neck were re-planned with AP version 9.10. Only one single cycle of plan optimization using one single template was allowed for AP. The dose to the planning target volumes (PTV’s; 3–4 dose levels), the organs at risk (OAR’s) and the effective working time for planning was evaluated. Additionally, two experienced radiation oncologists blind-reviewed and ranked 10 plans.

**Results:**

Dose coverage and dose homogeneity of the PTV were significantly improved with AP, however manually optimized plans showed significantly improved dose conformity. The mean dose to the parotid glands, oral mucosa, swallowing muscles, dorsal neck tissue and maximal dose to the spinal cord were significantly reduced with AP. In 64 % of the plans, the mean dose to any OAR (spinal cord excluded) was reduced by >20 % with AP in comparison to the manually optimized plans. In 12 % of the plans, the manually optimized plans showed reduced doses by >20 % in at least one OAR. The experienced radiation oncologists preferred the AP plan and the clinical plan in 80 and 20 % of the cases, respectively. The average effective working time was 3.8 min ± 1.1 min in comparison to 48.5 min ± 6.0 min using AP compared to the manually optimized plans, respectively.

**Conclusion:**

The evaluated automated planning algorithm achieved highly consistent and significantly improved treatment plans with potentially clinically relevant OAR sparing by >20 % in 64 % of the cases. The effective working time was substantially reduced with Auto-Planning.

## Introduction

Intensity modulated radiotherapy (IMRT) and volumetric modulated radiotherapy (VMAT) have been used for more than a decade and are now standard techniques for external beam radiotherapy treatment (RT). However, the inverse and computer-based planning approach involves multiple manual steps, which might influence the plan quality and consistency: planning objectives and constraints need to be manually adapted to the patient individual anatomy and tumor location, size and shape [1]. Additional help structures are frequently defined to further individualize and optimize the treatment plan on a patient individual basis resulting in an iterative process of IMRT and VMAT plan generation. Furthermore, the TPS operator needs to have profound knowledge and experience about the limitations of the treatment planning system and techniques, translating this into a prediction of the dose distribution, which can be achieved in each individual case. This method of manual optimization is time consuming, especially for complex cases such as head and neck carcinoma where multiple target dose levels are defined in close proximity to OAR’s.

In order to improve the overall plan quality and consistency, and to decrease the time required for planning, semi-automated planning algorithms have been developed [2, 3]. In the present study, we have evaluated a fully automated treatment planning system (TPS), Pinnacle Auto-Planning (Philips Radiation Oncology Systems), which uses an iterative algorithm based approach to automatically adapt objectives, constraints and dose shaping contours during the optimization process to achieve clinical goals. It was the aim of this study to evaluate Auto-Planning in a large cohort of head and neck cancer patients: treatment plans were compared with historical, clinically accepted VMAT treatment plans generated by Eclipse RapidArc planning (Varian Medical System, Palo Alto, CA). Additionally, time required for manual vs. Auto-Planning was compared.

## Methods and materials

### Automatic VMAT optimization

A new optimizer, Auto-Planning, was introduced in version 9.10 with the Pinnacle TPS (Philips Radiation Oncology Systems, Fitchburg, WI). It is a fully integrated module in the TPS, similar to the “manual” inverse optimizer module. Auto-Planning employs an iterative algorithm approach to reach and potentially surpass user defined clinical goals. During Auto-Planning, individual optimization goals, constraints and weights are automatically added and adjusted. The IMRT/VMAT optimizer is automatically run multiple times with adjustments being made during and between optimization runs. Additionally, Auto-Planning adjusts the priority of clinical goals based on their probability of being achieved. Therefore, Auto-Planning tries to mimic the decision making process of an experienced TPS operator. In addition to clinical objectives and priorities, Auto-Planning has a *compromise* setting to allow for sparing of serial organs such as the spinal cord over targets, and advanced settings allow the user to set global parameters such as priorities between targets and OAR’s, dose fall-off, maximum dose and cold spot management.

### Ethics approval and patient selection

All patients included into this study have given their approval to use their data for scientific research.

For this retrospective treatment planning study, 50 consecutive patients with diagnosis of primary mesopharynx, hypopharynx, oropharynx or larynx carcinoma patients treated in our department were enrolled. Each patient was treated with at least three dose levels: 70Gy or 69.63Gy, 60Gy and 54Gy within 35 or 33 fractions using a simultaneous integrated boost concept. Dose levels of 66Gy and 52Gy were additionally defined for six and 11 patients, respectively. The dose was normalized to the mean target dose of the highest-order planning target volume (PTV). The following critical structures were analyzed: parotids, spinal cord, brain stem, mandibular bones, oral mucosa, pharynx, dorsal neck tissue.

All patients were treated with the VMAT technique using two 360°- arcs. Manual VMAT treatment planning was performed using RapidArc planning in Eclipse treatment planning version 11.0.42. The treated plans were manually optimized for a 6-MV photon linear accelerator (Trilogy, TrueBeam or Edge; Varian Medical Systems). Clinically accepted and delivered treatment plans served as reference in this study. All plans were generated by experienced dosimetrists according to written protocols. All treatment plans were verified by a medical physicist and two radiation oncologist physicians before treatment for all cases.

### Retrospective planning study using Auto-Planning

Plans optimized with Auto-Planning utilized a single model where clinical objectives and priorities for each PTV and OAR are defined. The constraints for the target consist of a maximum dose allowed and a dose level to be achieved which corresponds to the dose to 98 % of the target volume. The clinical objectives for an OAR include dose-volume histogram, maximum dose and mean dose. Four priority levels can be defined for the OAR: *low, medium, high or constrain.* Depending on the patient-individual overlap of the OAR with the targets, the priority can be automatically adjusted from high to medium (>25 % overlapping), or to low (>50 % overlapping). It is possible for the user to over-ride automatic adjustment of individual priorities, but this is outside the scope of this investigation. In addition to priority, there is an additional *compromise* setting, which allows for individual OAR priority over targets (such as for spinal cord).

A model (pre-set of Auto-planning preferences and target & OAR objectives) for head and neck planning was created in Auto-Planning based on five head and neck cases. These five cases were not included in the set of patients used for the plan comparison. The Auto-Planning model was optimized by adjusting the clinical objectives and priorities until all five plans satisfied clinical target and organ at risk goals. All study plans were then optimized with the same model. The beam geometry for Auto-Planning was similar to the clinical plans using two 360°-arcs with collimator set to 5° and 355°. Ninety control points per arc were set for the Auto-Planning plans and 177 for the clinical plans. The ability to make individual adjustments to the model prior to running Auto-Planning was limited to the target doses in this study and was not allowed for OARs.

One treatment plan was generated for each patient using Auto-planning and only one optimization cycle was allowed. This plan was used for comparison with historical treatment plans.

Quality assurance for each clinical plan and for ten Auto-Planning plans was performed on a phantom (Delta4, ScandiDos AB, Uppsala, Sweden). All fields had to have a gamma value > 95 % with a distance to agreement of 3 mm and a dose difference of 3 %.

### Plan comparison

Dose–volume histograms (DVHs) were calculated for the PTVs and OARs for each plan. For comparison purposes, DVHs were normalized to the mean dose of the high dose PTV (70Gy or 69.63Gy over 35 or 33 fractions). Target dose distribution was evaluated according to the Paddick conformity index (CI) [4], the target coverage defined as the volume enclosed by the 95 % isodose line and the dose homogeneity index (HI) [1] defined as the ratio between the dose covering 5 % of the PTV volume to the dose covering 95 % of the PTV volume.

The dose to OARs was evaluated according to the mean dose for all OARs except for the spinal cord, where the maximum dose to 0.1 cm^3^ was evaluated.

In addition to DVH parameters evaluation, two experienced radiation oncologists blind-reviewed and ranked 10 randomized plans. They had to choose which plan they preferred.

### Planning time

The working time required by one highly experienced dosimetrist to generate a plan was measured for the last ten head and neck patients treated in our department. The time was measured for the manual optimized plans and for Auto-Planning. The planning time was defined as the effective working time required between the point where the target and OAR volumes are defined by the clinicians to the time were the plans is accepted by responsible clinicians. This included the time needed for the definition of the help structures and to re-adjust the constraints and help structures after each optimization. The time required for the optimization was not measured as it depends strongly on the number of plans running in parallel on the server.

### Statistics

Statistical analysis was performed using a paired *t* test. A p value of <0.05 was accepted as significant.

## Results

OAR objectives and priorities used for Auto-Planning are listed in Table 1. These settings were used for the optimization of all head-and-neck cases planned with Auto-Planning. No individualization was performed.

**Table 1:** Dose objectives used in Auto-Planning for the optimization of head and neck plans

	Constraint	Priority
Myelon	Dmax < 43Gy	Medium
Brainstem	Dmax < 48Gy	Medium
Parotid	Mean dose < 22Gy	High
Parotid	Mean dose < 15Gy	Medium
Oral mucosa	Mean dose < 20Gy	High
Oral mucosa	Mean dose < 12Gy	Low
Pharynx	Dmax < 45Gy	Medium
Pharynx	Mean dose < 20Gy	High
Pharynx	Mean dose < 15Gy	Medium
Plexus	Dmax < 61Gy	Medium
Mandibular bones	Dmax < 60Gy	Medium
Mandibular bones	Mean dose < 22Gy	Medium
Brain	Dmax < 48Gy	Medium
Brain	Mean dose < 20Gy	Medium
Dorsal neck tissue	Mean dose < 25Gy	Medium

### Target volumes

The average DVH parameters for the clinical and Auto-Planning plans for targets and OARs are shown in Table 2. The plans were normalized to the mean dose of the high dose PTV. Differences in dose homogeneity (HI) reached significance only for PTV 70Gy: HI was 1.066 ± 0.015 for Auto-Planning and 1.091 ± 0.017 for the clinical plans (*p* < 0.001). For PTV 60Gy and PTV 54Gy, HI was 1.098 ± 0.028 and 1.107 ± 0.018 for the Auto-planning plans and 1.103 ± 0.025 and 1.106 ± 0.021 for the clinical plans, *p* = 0.06 and p = 0.28.

**Table 2:** Dose-volume histogram parameters: comparison of clinical and Auto-Planning plans

	Clinical plan		AutoPlanning		*t*-test
	Mean	StDev	Mean	StDev	
Conformity index (PTV 70 Gy)	0.93	0.08	0.86	0.07	<0.01
Conformity index (PTV 54 Gy)	0.68	0.04	0.64	0.04	<0.01
Homogeneity index (PTV 70 Gy)	1.091	0.017	1.066	0.015	<0.01
Homogeneity index (PTV 60 Gy)	1.103	0.025	1.098	0.028	0.055
Homogeneity index (PTV 54 Gy)	1.106	0.021	1.107	0.018	0.279
Target coverage (PTV70Gy)	94.9	2.7	97.8	1.7	<0.01
Target coverage (PTV60Gy)	94.2	3.1	97.2	2.3	<0.01
Target coverage (PTV54Gy	93.9	3.0	95.2	2.7	<0.01
Myelon maximal dose (Gy)	42.7	3.6	41.1	2.6	<0.01
Mean ipsilat. parotid dose (Gy)	26.8	7.1	24.9	8.8	<0.01
Mean contralat. parotid dose (Gy)	21.4	5.3	19.4	5.9	<0.01
Mean swallowing muscles dose (Gy)	33.5	8.7	30.4	7.7	<0.01
Mean oral mucosa dose (Gy)	29.2	5.2	27.5	6.0	<0.01
Mean mandibular bones dose (Gy)	34.9	4.4	31.6	6.1	<0.01
Mean dorsal neck tissue dose (Gy)	28.2	5.0	24.8	3.7	<0.01

Abbreviations: *PTV* planning target volume

The target coverage was significantly improved with Auto-Planning by 2.9, 3.0 and 1.3 % for the PTV 70Gy, PTV 60Gy and PTV 54Gy (*p* < 0.01). However, the CI decreased significantly with Auto-Planning (*p* < 0.001) for the PTV 70Gy and for the PTV from 0.93 to 0.86 and from 0.68 to 0.64.

### Organs at risk

Comparison of OAR sparing is summarized in Table 2. Both Auto-Planning and clinical plans were able to keep the maximum spinal cord dose below our tolerance of 45Gy for each patient. Auto-Planning further reduced the maximal dose to the spinal cord by 1.6Gy in comparison to the clinical plans (*p* < 0.01).

**Fig. 1:**
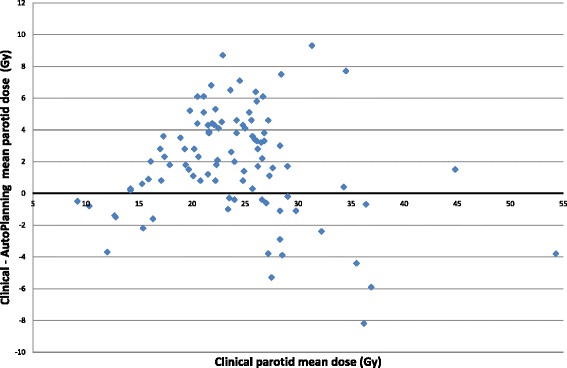
Mean parotid dose difference as a function of the clinical mean parotid dose. If the values are positive, Auto-Planning reduced the parotid mean dose. If the values are negative, the parotid mean dose was reduced with the clinical plan

The mean doses to the ipsilateral and contralateral parotid were reduced with Auto-Planning by 1.9Gy and 2.0Gy respectively (*p* < 0.01), see Table 2. The largest reduction of the mean parotid dose with Auto-Planning was observed for clinical plans having a mean parotid dose between 17Gy and 27Gy, see Fig. 1. In contrast, the parotid mean dose increased on average with Auto-Planning when the mean parotid dose was below 15Gy or above 35Gy for the clinical plans. The parotid mean dose was reduced by more than 10 and 20 % with Auto-Planning in 49 and 19 % of the cases, respectively. Contrarily, the parotid mean dose was increased with Auto-Planning by 10 and 20 % in 11 and 2 % of the cases, respectively (Table 3).

**Table 3:**
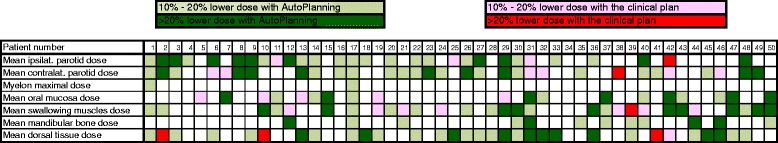
Dose difference between the clinical and the Auto-Planning plans

Each column represents the results for a patient. In light green, respectively dark green, Auto-Planning reduced the organ dose by more than 10 %, respectively 20 % in comparison to the clinical plan. In pink, respectively red, the clinical plan reduced the organ dose by more than 10 %, respectively 20 % in comparison to Auto-Planning

**Fig. 2:**
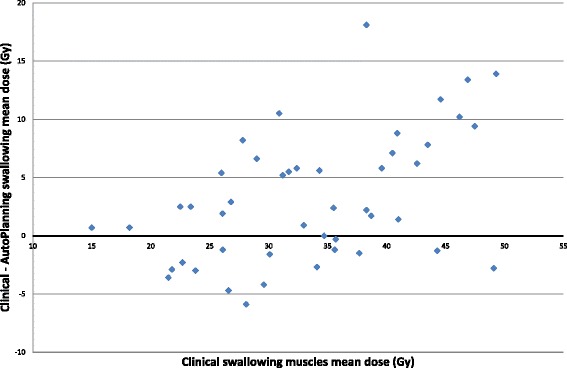
Mean swallowing muscles dose difference as a function of the clinical mean swallowing muscles dose. If the values are positive, Auto-Planning had a better dose sparing of the swallowing muscles. If the values are negative, the clinical plan had a better dose sparing of the swallowing muscles

**Fig. 3:**
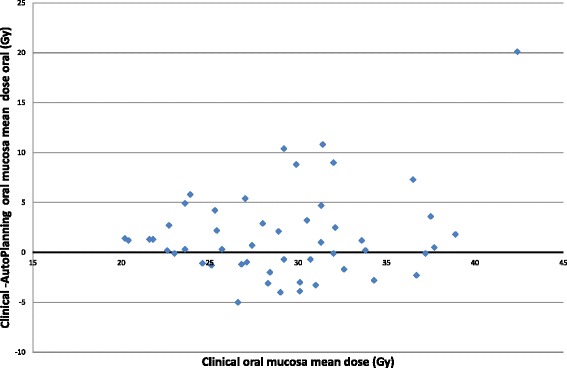
Mean oral mucosa dose difference as a function of the clinical mean oral mucosa dose. If the values are positive, Auto-Planning had a better dose sparing of the s oral mucosa. If the values are negative, the clinical plan had a better dose sparing of the oral mucosa

The mean dose to the swallowing muscles, oral mucosa, mandibular bones and dorsal neck tissue was significantly reduced with Auto-Planning by 3.1Gy, 1.7Gy, 3.3Gy and 3.4Gy (*p* < 0.01), respectively (see Table 2, Figs. 2 and 3). In ten, seven, four and ten cases the mean dose to the swallowing muscles, oral mucosa, mandibular bones and dorsal neck tissue was reduced by more than 20 % with Auto-Planning, see Table 3. In contrast, the clinical plans showed lower mean doses to the swallowing muscles by >20 % in one case and for the dorsal neck tissue in three cases.

In 32 plans (64 %), Auto-Planning was able to reduce at least one OAR (spinal cord excluded) by more than 20 %. In contrast, the clinical plans showed reduced doses to at least one OAR by more than 20 % in six cases (12 %), see Table 3.

The results from the blind ranking performed by two experienced radiation oncologists showed that Auto-Planning plans were preferred in 80 % of the cases and in 20 % of the cases, the clinical plans were preferred.

### Planning time

The effective working time required after volume definition by the clinicians to the end of the optimization, when the plan is ready to be checked by the clinicians, was measured for the last ten patients planned. 3.8 min ± 1.1 min ranging from 2.45 to 6.33 min were required for Auto-Planning compared to 48.5 min ± 6.0 min for the clinical plans ranging from 42.0 to 56.5 min.

## Discussion

In this comparative study, plans manually optimized with Eclipse were compared to plans automatically optimized with Pinnacle. Both systems are using different optimizer algorithms which potentially influence change the results. Nevertheless, planning challenges involving experienced operators have shown that both systems were able to achieve similar dose distribution [5, 6]. Therefore, we do not believe that the results are biased due to the use of two different optimization algorithms.

Results of our study show that Pinnacle Auto-Planning, an automated iterative planning algorithm, achieved highly consistent treatment plans: target coverage and homogeneity were significantly improved at the expense of dose conformity compared to clinically accepted historical plans. However, dose differences for all target volumes were small and are unlikely to be clinically relevant. Additionally, Auto-planning achieved a significant reduction of all OAR dose. Most importantly, Auto-Planning was able to reduce the dose to at least one OAR by more than 20 % in 64 % of the cases.

This finding of improved OAR sparing by a potentially clinically relevant amount could be explained by sub-optimal constraints being used in the historical manual planning cohort. Plan optimization for head and neck is one of the most complex scenarios due to the complexity of the tumor shape, dose levels and location. Targets with multiple dose levels are defined in close proximity to or as overlapping OARs making the optimization a complex and time consuming process. The result will strongly depend on the skill set of the TPS Operator when setting the correct help structures, optimization objectives and constraints.

In 19 % of the plans, the mean dose to one of the parotids was reduced by more than 20 % with Auto-Planning. This reduction of the mean dose is expected to be clinically relevant and reduce xerostomia [7]. The normal tissue complication probability for the parotid glands reported by Eisbruch et al. is described as a continuous and monotonously increasing function of the mean dose with a steep gradient between 20Gy and 30Gy [8]. Any significant reduction of the mean dose in this range may thus be of clinical benefit. The mean parotid dose increased with Auto-Planning for clinical plans having a dose larger than 35Gy. For these cases, the targets were largely overlapping with the parotid. If this overlap is larger than 25 and 50 %, the priority was automatically reduced to medium or low with Auto-Planning. This could explain the higher mean parotid dose with Auto-Planning in cases where a mean dose greater than 35Gy was observed.

A significant correlation between the mean swallowing muscle dose and complications such as dysphagia has been observed [9]. A steep dose-effect relationship, with an increase of the probability of dysphagia of 19 % with every additional 10Gy, was established [10]. We observed that Auto-Planning could be clinically beneficial for patients in respect to the dose delivered to the swallowing muscles. Indeed, in 20 % of the cases, Auto-Planning was able to reduce the swallowing muscles mean dose by more than 20 % which correspond to a dose reduction of 10.7Gy.

Highly consistent and improved plan quality in a relevant proportion of the cases compared to our historical plans was not only observed in DVH parameters. Indeed, radiation oncologists specialized in head and neck cancer preferred Auto-Planning plans compared to the clinical plans in 80 % of the cases; however they simultaneously stated that results were close and both planning methods achieved clinically acceptable plans in all patients. This result is even more surprising as only one single Auto-Planning head and neck model—pre-set of optimization parameters—was used in Auto-planning and only one single optimization cycle was allowed. One could therefore speculate that further optimization of the Auto-Planning approach might be possible by 1) more customized optimization models and by 2) the use of more than one optimization cycle. By increasing the number of optimization cycles, the planner would be able to change the targets and/or OARs objectives in order to modify the dose distribution previously calculated. This would lead to a more patient specific dose distribution. Further analyses are ongoing to test these hypotheses. Besides potentially improved outcome on a patient-individual level and on an institutional level, such automated planning approaches are considered to be especially useful within the context of multi-center trials to standardize and homogenize planning and plan quality.

Other automated planning approaches have been discussed elsewhere [9, 10]. One method is based on a previously generated site-specific plan library: the individual patient is compared to the patient population in library of plans and similarity measures are used for selection of planning optimization parameters [2, 9, 11]. This compares patient-individual DVH parameters to the database and estimates plan quality in comparison to an overall patient population. However, the application of the library is restricted to patients with identical or very similar planning objectives (number of fractions, target and OAR doses) [9]. Also, a larger number of plans are required for de-novo generation a robust library, which is challenging for smaller institutions and/or rare indications. Finally, since the knowledge of the previous plans is used to generate the objectives, the newly generated plan quality inevitably depends on the quality of the plans building the database [11]. Non-optimal plans entered in the database may degrade results with the plan library approach.

Multicriteria optimization (MCO) approaches [6–8] have been proposed for real-time assessment of the trade-off between different clinical goals. MCO allows to navigate towards a Pareto optimal plan in a database of automatically generated plans and thereby balance between targets and between organs at risk. One limitation of the MCO approach is the increased time to create the library of treatment plans before dose evaluation can be performed, although this can be overcome by faster dose calculations [12]. The MCO approach also requires significant individual user experience and interaction to determine the Pareto optimal plan, which may become especially challenging in head and neck cases were multiple targets and organs at risk then need to be balanced. In addition, if the MCO generated library of plans is fluence based, the Pareto optimal plan will not be deliverable, and further fluence segmentation is required.

## Conclusion

In conclusion, our study shows that the Pinnacle Auto-Planning approach achieves highly consistent treatment plans for complex head-and-neck cancer patients: dose distributions of the target volumes were similar between the automated planning approach and manual planning. OAR sparing was significantly improved using Auto-Planning and potentially relevant improved OAR sparing was observed in more than half of the patients. Furthermore, experienced radiation oncologists preferred the dose distributions from Auto-Planning in comparison to those from the manually generated plans in 80 % of the cases. Human resources required for treatment planning was substantially reduced and made independently from the experience of the treatment planner.
